# First whole-genome sequence of *Mycobacterium avium* subsp. *silvaticum* isolated from a diseased Egyptian goose (*Alopochen aegyptiaca*)

**DOI:** 10.1186/s12864-025-11893-3

**Published:** 2025-08-11

**Authors:** Stefanie A. Barth, Martin Peters, Sascha Mormann, Petra Möbius, Sten Calvelage, Hanka Brangsch

**Affiliations:** 1 Friedrich-Loeffler-Institut– Federal Research Institute for Animal Health (FLI), Institute of Molecular Pathogenesis (IMP), Jena, Germany; 2 National Reference Laboratory for Bovine Tuberculosis, Jena, Germany; 3 Chemisches- und Veterinäruntersuchungsamt Westfalen (CVUA Westfalen), Arnsberg, Germany; 4 Friedrich-Loeffler-Institut - Federal Research Institute for Animal Health (FLI), Institute of Diagnostic Virology (IVD), Greifswald, Isle of Riems Germany; 5 Friedrich-Loeffler-Institut - Federal Research Institute for Animal Health (FLI), Institute of Bacterial Infections and Zoonoses (IBIZ), Jena, Germany

**Keywords:** Egyptian Goose (*Alopochen aegyptiaca*), *Mycobacterium avium* subsp. *silvaticum*, Avian tuberculosis, Whole genome sequencing, Bacterial culture, Mycobactin cluster

## Abstract

**Background:**

Among the non-tuberculous mycobacteria, *Mycobacterium (M.) avium* are important pathogens for humans and/or animals. Currently, there are four *M. avium* subspecies: subsp. *hominissuis (Mah)*, subsp. *paratuberculosis (Map)*, subsp. *avium (Maa)*, and subsp. *silvaticum (Mas)*. While sufficient data is available for the first three mentioned, only few reports exist on the isolation, epidemiology and even less on the genetic equipment of *Mas*.

**Results:**

Here, *Mas* was isolated from an Egyptian goose that died of avian tuberculosis. Subspecies identification was based on the presence of IS*901* and IS*1245* as well as Mycobacterial Interspersed Repetitive Units-Variable Number Tandem Repeat analysis demonstrating *Mas* specific profile INMV99 profile.

During cultural isolation, *Mas* showed preference for media with mycobactin supplementation but was not limited to mycobactin-containing media.

A closed genome sequence was assembled using short- and long-read sequencing technology. The genome sequence consisted of one circular chromosome of 4.84 Mb (GC content 69.3%) and no plasmid. It was highly similar to the only other available *Mas* sequence (ANI 99.98%, GGDC 99.7%) and eight *Maa* sequences (ANI ≥99.88%, GGDC ≥98.9%), although all *Maa* genomes were larger (approx. 5 Mb).

*In silico* prediction of the metabolic pathways and gene content found that all *Maa* but no *Mas* should be able to synthetize ergothioneine and the carotenoid neurosporene. The analysis of the mycobactin cluster *mbt*-1 made it obvious that in *Mas *two of the eleven *mbt* genes (*mbtB* and *mbtE*) were probably dysfunctional due frameshift-based disruptions.

**Conclusions:**

The first complete, high quality, closed genome sequence of a *Mas* isolate closes a knowledge gap. Even if the collection of further genome sequences is considered necessary, the now existing data set already enables a deeper analysis of *M. avium*. The found differences in the *Mas* gene content compared to the closest relative *Maa* seem to be stable and independent of spatial (France, UK, Germany) and temporal (>40 years) differences on their isolation. These data thus call into question the demand for merging the two subspecies *Maa* and* Mas* into one, but further genome sequences from other *Mas* strains are needed to answer this question conclusively.

## Introduction

Egyptian geese (*Alopochen aegyptiaca*) originate from sub-Saharan Africa and Egypt, but in recent decades they have invaded central Europe, in particular the Netherlands, Belgium, Germany and England, among other countries [1, 2]. Since 2009, an established neozotic population exits in Germany [3–5]. The animals live nearby freshwater systems, like rivers or lakes [6].

Waterfowl are the main reservoir hosts for avian influenza (AI) viruses, including highly pathogenic avian influenza (HPAI) virus, which can cause high mortality rates with massive economic losses when commercial flocks are affected [7]. HPAI can also infect mammals, including humans. Thus, the EU legislation and subsequent German legislation [8] require active and passive monitoring programs in poultry to obtain early evidence of unusually high mortality rates and disease outbreaks, particularly in waterfowl species. As part of the monitoring program, any waterfowl found dead has to be submitted for necropsy. According to the German Animal Disease Reporting System (TSN) in 2021 and 2022 AI was detected in four dead Egyptian geese in Germany. Three of them were tested positive for HPAI strain subtype H5N1 [9].

However, other causes of death in birds are also recorded, based on the regular necropsies carried out in the search for AI. This includes, among others, avian tuberculosis (avTB), another common disease in poultry. The causative agents of avTB are *Mycobacterium* (*M.*) sp., in particular *M. avium* subspecies *avium* (*Maa*) or subspecies *hominissuis* (*Mah*) and *M. genavense* (*Mg*) [10, 11]. The species *M. avium* additionally includes subspecies *silvaticum* (*Mas*) and subspecies *paratuberculosis* (*Map*) [12, 13]. The latter, *Map*, is an important pathogen in ruminants, causing Johne’s disease [14], while *Mas* is predominantly found in wild birds and mammals (e.g. red deer, wild boar), but not the environment [15]. In contrast, *Mah* is a ubiquitous bacterium occurring in soil and water, that causes infection in a wide range of hosts, including mammals and birds, but usually after immune suppression in the host [16, 17].

Despite these differences in host adaption and clinical outcome, the four *M. avium* subspecies are genetical highly similar, so that a taxonomic revision into one species has been discussed [18]. Among those four subspecies, the most homologous are *Maa* and *Mas* [18, 19]. Using common typing methods, e.g. PCR detection of the presence of specific insertion elements (IS*1245*, IS*901*, IS*902*) or Sanger sequencing of 16S rRNA, *hsp65* and *rpoB* fragments, *Maa* and *Mas* are not distinguishable [20]. In 2015, a subspecies-specific real-time PCR with a high-resolution melting curve analysis was published that was able to discriminate the subspecies [21]. To date, no complete *Mas* genome sequence is available, so knowledge of possible subspecies-specific genomic determinants is very limited.

In the case presented here, a deceased Egyptian goose was submitted for necropsy according to the GeflPestSchV [8]. Massive pathological changes were found in the internal organs, consistent with the clinical picture of avTB. The subsequent isolation of the pathogen led to the identification of *Mas*, a very rarely detected subspecies. Here, we describe the clinical picture and provide the first complete genome sequence of a *Mas* isolate including an in-depth genomic characterisation of the isolate.

## Materials and methods

### Clinical specimen

In 2022, an adult male Egyptian goose (2.1 kg) was found dead in the Märkischer Kreis district in North Rhine-Westphalia, Germany, and was sent for necropsy according to the passive wild bird monitoring prescribed by German legislation [8].

The post-mortem examination of the bird was done under appropriate safety levels. Tissue sections from liver and spleen were taken for microbiological examination. Further, parts of the tissue samples were fixed in 10% neutral buffered formalin, embedded in paraffin wax, sectioned at 4 μm and stained with hematoxylin and eosin (H&E) and Ziehl Neelsen stain for histological examinations.

### Microbiological examination

Tissue sections from liver and spleen were homogenized and decontaminated using N-acetyl-L-cystein-NaOH (final concentration 1%, 25 min, shaking). After two washing steps, the suspension was cultured on the media regularly used in our laboratory for mycobacterial diagnostic approaches, if not *Map* is suspected. This included Löwenstein-Jensen/glycerol/PACT (polymyxin B, amphotericin, carbenicillin, trimethoprim) and Coletsos/PACT agar slants (both Artelt-Enclit, Borna, Germany) as well as Middlebrook 7H9/glycerol/OADC (oleic acid, albumin, dextrose, catalase) broth (in house). Cultures were incubated at 37 °C (+/- 2 °C) up to 12 weeks and weekly controlled for suspicious colonies.

For the analysis of growth characteristics and mycobactin J dependence of strain 22MA0766, the following additional culture media were used: Herrold’s egg yolk/ANV (amphotericin, nalidixic acid, vancomycin)/mycobactin J agar (Becton Dickinson, Heidelberg, Germany), Heym 3/glycerol/PACT agar (in house), Middlebrook 7H9/glycerol/OADC/mycobactin J broth (in house), as well as Kirchner/PACT broth (Himedia, Modautal, Germany).

### Molecular genetic examinations

#### Avian influenza virus testing

Exclusion testing for avian influenza virus was performed using an accredited real-time RT-PCR procedure on an Mx3005P (Agilent Technologies, Santa Clara, CA, USA) thermocycler. RNA was extracted from combined cloacal throat swab material using the IndiMag Pathogen Kit on the IndiMag 48 automated magnetic bead-based extraction workstation (both INDICAL BIOSCIENCE GmbH, Leipzig, Germany). The extract was subsequently analysed for influenza A virus-specific nucleic acid in a duplex assay together with β-actin as an internal control using the virotype Influenza A RT-PCR Kit (INDICAL BIOSCIENCE GmbH, Leipzig, Germany) according to the manufacturer’s specifications.

#### Mycobacterial diagnostics

DNA was extracted from 0.2 g tissue parts of liver and spleen using the DNeasy blood and tissue kit (QIAGEN, Hilden, Germany). The extract was tested for the presence of DNA specific to *M. avium* and *M. genavense* using two real-time PCR approaches (rtPCRs) [22–24]. Both rtPCRs were conducted as duplex rtPCR with parallel detection of host’s β-actin gene in a Taqman Gene Expression Mastermix (ThermoFisher Scientific, Darmstadt, Germany).

An heat-ultrasound-lysate of putative mycobacterial colony material was tested by conventional PCR for the presence of the *M. avium* subspecies-specific insertion elements *IS*1245 [25] and *IS*901 [26]. Subsequently, the *M. avium* isolates were molecularly characterized using Mycobacterial Interspersed Repetitive Units-Variable Number Tandem Repeat (MIRU-VNTR) analysis. According to Thibault, Grayon [27] the loci VNTR 292, X3, 24, 47, 3, 7, 10, and 32 were included and resulting patterns were compared with the database MAC-INMV-SSR [28].

### Whole genome sequencing (WGS)

#### DNA sequencing

For high molecular weight DNA preparation, isolates were grown on Herrold’s egg yolk agar with mycobactin J (Becton Dickinson, Heidelberg, Germany) for six weeks. The DNA was extracted using the cetyltrimethyl-ammonium bromide (CTAB) method [29] with an additional RNase A digestion in combination with the lysozyme digest and additional washing steps after the alcoholic precipitation of the DNA. Short-read sequencing of the DNA was done by Illumina NovaSeq technology using a commercial sequencing service (Eurofins Genomics, Konstanz, Germany). Long-read sequencing was performed on a PromethION P2 solo device (Oxford Nanopore Technologies Ltd, Oxford, United Kingdom) using R10.4.1 flow cells (FLO-PRO114M). The libraries were prepared with the Rapid Barcoding Kit SQK-RBK114-24 (Oxford Nanopore Technologies Ltd, Oxford, United Kingdom) and run for 48 h. Guppy v6.5.7 (Oxford Nanopore Technologies Ltd) with the high accuracy model dna_r10.4.1_e8.2_400bps_5khz_hac_prom was used for basecalling. Long-read sequencing quality was assessed using Nanoplot v1.42.0 [30].

#### Post-sequencing processing of raw data

Illumina and Nanopore reads were screened for contaminations using Kraken2 v2.1.1 [31]. The Bacterial ONT data pipeline v1 (https://gitlab.com/ChristineThms/bacteriapipeline) was used for sequencing data processing. The workflow comprised prechop and filtlong for trimming and filtering of Nanopore reads, respectively, as well as trimmomatic for Illumina read trimming. Flye and Unicycler were chosen in the workflow for generating two *de novo* genome assemblies based on long-read first and short-read first approaches, respectively. Both assemblies were polished by polyPolish using the Illumina reads. The contigs were reoriented by dnaapler to start with *dnaA*, when possible. Assembly statistics were assessed by QUAST.

For assessing the comparability of both assembly approaches, the assemblies were aligned by Mauve v2.4.0 [32] using the progressiveMauve algorithm. One assembly was chosen for further investigation.

The raw sequencing data and the assembly is made publicly available by deposition in the European Nucleotide Archive (ENA) under the project number PRJEB87763.

#### Genome characterization and genotyping

Completeness and contamination status were checked using BUSCO v5.7.1 with the actinobacteria_class_odb10 database (creation date: 2024-01-08) [33] and CheckM v1.2.3 [34]. The genome was annotated by Bakta v1.9.4 with database 5.1 (full) [35] and visualized by GenoVi v0.4.3 [36], which also predicted the Clusters of Orthologous Groups (COGs) for the predicted translation products. Genes related to antimicrobial resistances were identified using the Comprehensive Antibiotic Resistance Database (CARD) [37] in conjunction with ABRicate v1.0.1 (https://github.com/tseemann/abricate). Further, the Virulence Factor Database (VFDB) [38] was used for identification of virulence-associated genes (accessed online at 07.02.2025). IS element detection was done with ISEScan v1.7.2.3 [39] and the results were confirmed by browsing the annotation file. Additionally, IS*901* (acc. no. X59272.1) and IS*902* (acc. no. X58030) elements were searched using ABRicate with both sequences as queries. The sequences were extracted, aligned and compared using Geneious Prime^®^ v2021.0.1 (GraphPad Software, Boston, MA, USA).

For assessing the similarity of the investigated strain to other *M. avium* species, all publicly available *M. avium* genomes were downloaded (accession date: 21.02.2025) from RefSeq and checked by QUAST v5.0.2 [40]. Genomes with more than 200 contigs and an N50 < 10,000 bp were excluded from the analysis for quality assurance. Further, NCBI’s short read archive was browsed (accession date: 24.02.2025) for *Mas* sequencing data. Available data was downloaded and assembled by Shovill v1.0.4 (https://github.com/tseemann/shovill) using SPAdes. The average nucleotide identity (ANI) was calculated using fastANI [41] as implemented in ANIclustermap v1.4.0 (https://github.com/moshi4/ANIclustermap); a tool that clustered the fastANi output matrix by scipy’s UPGMA method and drew an ANI clustermap by seaborn v0.13.2 [42]. Trees were visualised by FigTree v1.4.3 (http://tree.bio.ed.ac.uk/software/figtree/).

Additionally, the genomic identity between the studied strain and other *Maa*/*Mas* strains was determined using the Genome-to-Genome Distance Calculator (GGDC) 3.0, online (accession date: 26.02.2025), that estimated the DNA-DNA hybridization percentage based on the sum of all identities found in high-scoring segment pairs (HSPs) divided by overall HSP length (formula 2) [43].

Complete genomes were aligned by Mauve as described above for inspecting genomic rearrangements. Also, metabolic pathways in *Mas* and *Maa* strains were predicted by gapseq v1.4.0 [44] and differences in gene content was investigated using Panaroo v1.4.2 [45].

#### In silico MLST and MIRU-VNTR

The MLST analysis for *Mas* and *Maa* strains is based on nucleotide variations in the DNA sequence of the following conserved genes: *recF*, *aspB*, *gnd1*, *lipT*, *pepB*, and a putative esterase, referred to as ‘*est*’ [46]. The sequences of these genes in the genome of interest (22MA0766) were compared in silico with the corresponding sequences of the reference strain *M. avium* 104 (NC_008595.1) using the software Geneious Prime^®^ v2021.0.1 (GraphPad Software, Boston, MA, USA). Single nucleotide polymorphisms (SNPs) at specific variable positions in the respective genes were detected and results assigned to defined specific sequence alleles [46, 47]. The specific allele numbers for the target genes were concatenated to allele profiles and assigned to the respective sequence type (ST), also established previously [46, 47].

Additionally, in silico MIRU-VNTR analysis was performed detecting the number of repeats in different loci sets defined by Ronai et al. [15] and Thibault et al. [27] by using the in_silico_PCR script by Egon Ozer (https://github.com/egonozer/in_silico_pcr).

#### Characterisation of the *mbt* cluster

A protein sequence database comprising the Mbt proteins of *Mycobacterium avium* required for mycobactin synthesis was created, based on the findings of Chavadi et al. [48]. Three representative genomes of *Mah* (GCF_000014985.1), *Map* (GCF_000007865.1) and *Maa* (GCF_001683455.1) were annotated by Bakta. The predicted translation products of these representative genomes together with 22MA0766 were screened using DIAMOND v2.1.8.162 [49]. The detected proteins were compared by alignment with MAFFT v7.520 [50] and open reading frames were visualized using the R package gggenes v0.5.1 [51].

## Results

### Necropsy

**Fig. 1:**
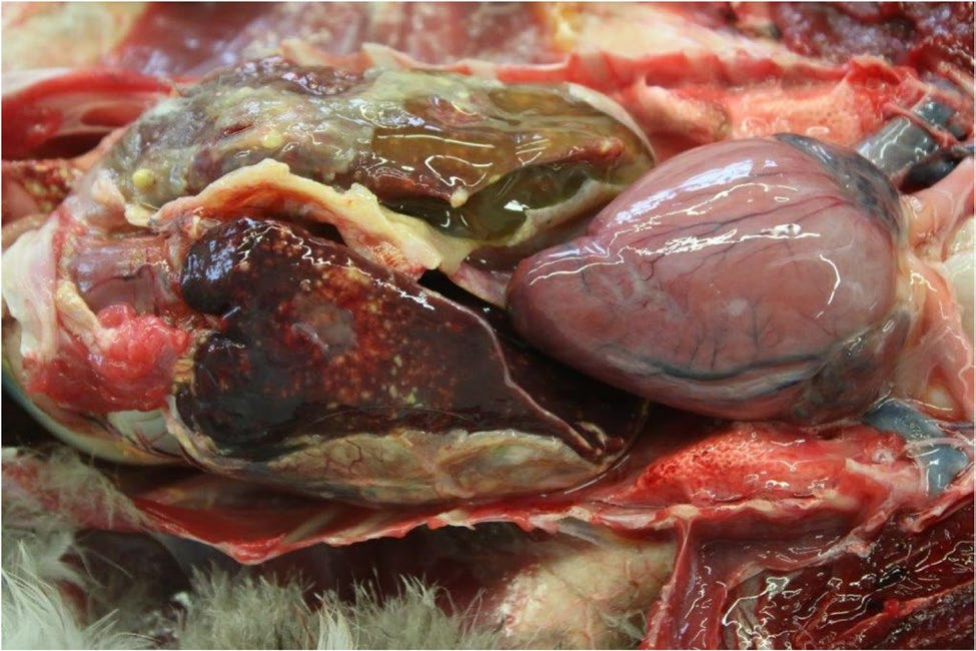
Disseminated avian tuberculosis in an Egyptian goose. Granulomatous nodules present in inner organs (e.g. liver, spleen). © CVUA Westfalen

The bird was in a poor nutritional state and had a diffuse granulomatous coelomitis with severe hepatosplenomegaly. The liver was covered with a layer of fibrinopurulent exudate and showed confluent yellow-white spots of variable size (Fig. 1).

**Fig. 2:**
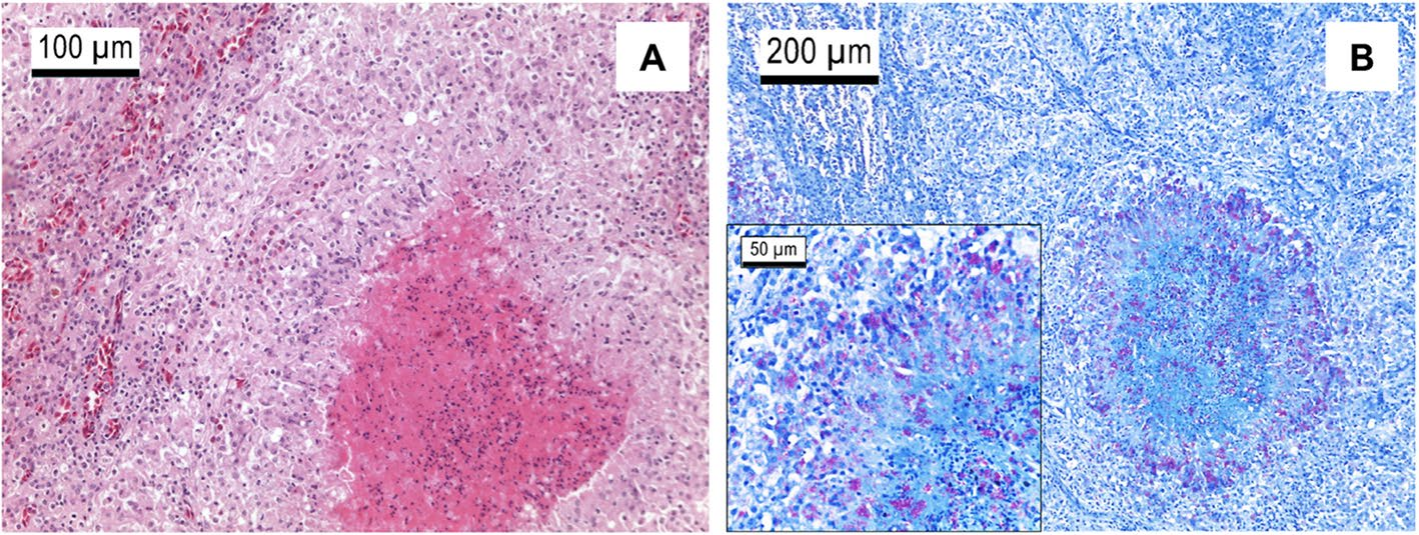
Histological examination of liver tissue of an Egyptian goose. **A** Liver, H&E stain: mycobacterial granuloma. **B** Liver, Ziehl-Neelsen stain: multibacillary granulomatous mycobacteriosis. © CVUA Westfalen

Histologically, there were multifocal granulomas with necrotic centres surrounded by multinucleated macrophages and histiocytes (Fig. 2A). Ziehl Neelsen stain revealed abundant intralesional extra- and intracellular acid-fast bacteria (Fig. 2B).

### Strain isolation and identification

Nucleic acid extracts from swap and tissue samples, respectively, were tested negative for Influenza A virus, but positive for *M. avium* by real-time PCRs. After two weeks of cultivation, the 7H9 broth culture inoculated with tissue from the liver showed bacterial growth while on egg-based agar slants no growth was detected even after eight weeks of incubation.

By conventional PCR, the presence of IS*1245* and IS*901* was shown, leading to the presumptive diagnosis of an *Maa/Mas* infection. MIRU-VNTR analysis yielded the pattern 2-4-1-3-1-1-1-7, corresponding to profile INMV99, which is also found in *Mas*, according to the database [28].

**Fig. 3:**
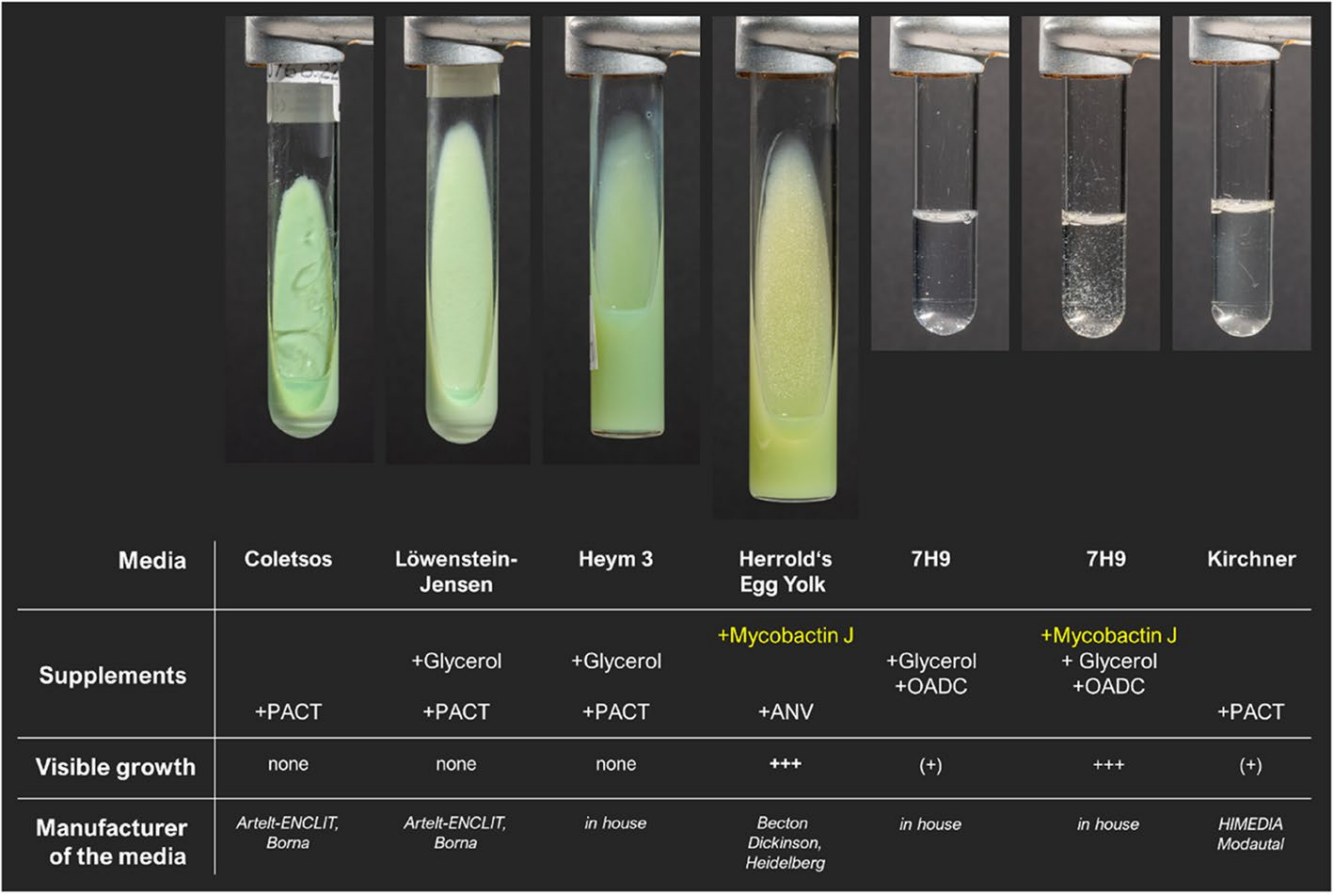
Growth characteristics of strain 22MA0766 after 20 days at 37°C. The isolate was sub-cultivated on Coletsos, Löwenstein-Jensen, Heym 3 and Herrold’s Egg Yolk with mycobactin J agar slants, as well as in 7H9, 7H9 with mycobactin J, and Kirchner broth. PACT, polymyxin B, amphotericin, carbenicillin, trimethoprim; ANV, amphotericin, nalidixic acid, vancomycin; OADC, oleic acid, albumin, dextrose, and catalase supplement (Becton Dickinson). © FLI/IMP

After sub-cultivation of the strain, named 22MA0766, growth was only visible on egg-based agar containing mycobactin J or in egg-free broth cultures (Kirchner or 7H9 broth). In 7H9, the addition of mycobactin increased the growth rate dramatically and reduced the incubation time (Fig. 3 and Supplemental Figures S1 and S2).

### WGS analysis

#### Sequencing quality and genome characterization

Long-read sequencing yielded 1,160,705 reads, equalling 2,634,442,808 bases and a coverage of 418x, with a median read length of 1,303 bp and median quality of 19.9. The sequencing depth achieved by Illumina was 969x and 99.2% of the reads was classified as originating from a *Mycobacterium avium* complex bacterium, which was in the same range as for the long reads (98.5% of reads).

As the BONT pipeline offered the possibility to use multiple assemblers in parallel, we chose flye and Unicycler to see if one approach (long-read first or short-read first, respectively) is better suited for *Mas* genome assembly or if they gave identical results. With both tools one complete contig of almost identical size (~ 4.841 Mb) was constructed. When aligned, both assemblies were identical in structure. Thus, the flye assembly was chosen for further investigation, although the Unicycler assembly was of identical quality.

**Fig. 4:**
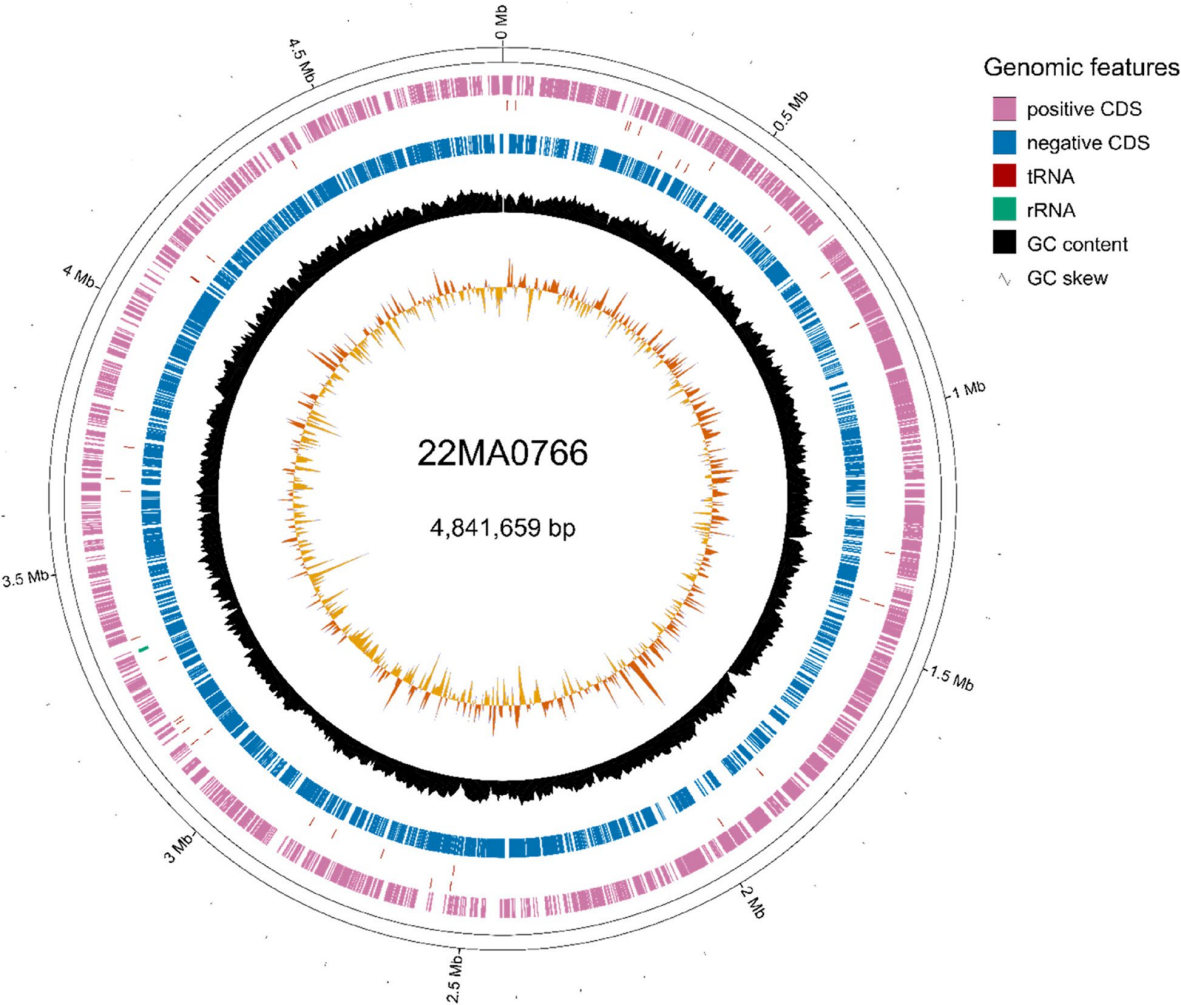
Genome representation of strain 22MA0766

The genome of 22MA0766 consisted of a single circular chromosome of 4,841,659 bp with a GC content of 69.29%. Overall, 4,540 coding sequences were identified, with 27 pseudogenes and 127 hypothetical coding regions. Further, 46 tRNA, one tmRNA, three rRNA and 15 ncRNA sequences were detected (Fig. 4). Screening for antimicrobial resistance-related genes yielded only four hits: *efpA*, *mtrA*, *rbpA*, and a variant of *rpoB2*. Additionally, 209 mycobacterial virulence-associated genes were detected (Supplementary Table S1).

No contaminating sequences were detected by both, BUSCO and CheckM. The high percentage of detected actinobacterial marker genes (689 of 693; 99.61%) and complete, single-copy universal orthologous genes (BUSCOs) (353 of 356; 99.2%) confirmed the completeness of the genome assembly.

Overall, 50 transposable elements were detected in the genome of 22MA0766. Of these, 25 belonged to the IS*110* family, 16 of which were identified as IS*901*/IS*902*. Interestingly, the database search for both of these elements did not yield perfect hits for any of the two elements, as the identity was between 99.25–99.6%. Thus, the sequences of the corresponding transposase-coding regions were extracted and compared. In one instance, the transposase gene was truncated at the 5’ terminal end. Fourteen transposase genes were identical and showed 18 nucleotide positions that were either specific for IS*901* or IS*902* (Supplementary Table S2), i.e. 11 IS*901*- and seven IS*902*-specific positions. A single transposase gene showed an additional mutation at position 29 (G to T). Besides IS110 family elements, also one IS*1245*, two IS*1311*, three IS*1601*, nine IS*Mav1*, two IS*Mav2*, one IS*481* family, three transposase *Mu* and four transposases of unknown identity were detected.

#### In silico identification and confirmation of *Mas* subtype by marker sequences

As the two other *Mas* strains, strain 22MA0766 also belonged to the known *Mas* MLST sequence type ST26 with the allelic profile: 8-3-7-6-6-4, based on sequence variances in MLST target genes *recF*, *aspB*, *gnd1*, *lipT*, *pepB*, and ‘*est*’ (Table 1). Further, the INMV profile was confirmed in silico as INMV99, which was also found for *Mas* strain MASMRI001. However, the *Mas* strains differed in two MIRU-VNTR loci set by Ronai et al. (2016) [15], whereas 22MA0766 only differed in a single locus of this panel from two *Maa* strains of INMV100 (RCAD0278, Jb0769).

**Table 1:** *In silico *typing of *Maa* and *Mas* strains using MIRU-VNTR, MLST, and variable regions

**NCBI RefSeq or SRA accession no.**	**Strain**	**Sub-spe-cies**	**Host**	**Origin**	**MIRU-VNTR analysisNumber of repeats per strain and locus**		**MLST analysis allele profile/sequence type**	**Duplex HRM rtPCR**^**4**^	
					**Loci set by Ronai et al. 2016** [15]^**1**^	**Loci set by Thibault et al. 2007** [27]^**2**^**/INMV profile**	** Turenne et al., 2008** [46]; **Kolb et al., 2014** [47]^**3**^	** Ronai et al., 2015** [21]	
this study	22MA0766	*Mas*	goose	GER	1-5-9-5-1-7-3-1	2-4-1-3-1-1-1-7/INMV99	8-3-7-6-6-4/ST26	ATTA	ACCT
ERR266517	MASMRI001	*Mas*	n.k.	UK	1-5-5-5-1-7-2-1	2-4-1-3-1-1-1-7/INMV99	8-3-7-6-6-4/ST26	....	....
GCF_000504975.1	ATCC49884	*Mas*	pigeon	FRA	1-5-5-5-1-NA-2-1	2-2-1-3-1-1-1-NA/NA	8-3-7-6-6-4/ST26	....	....
GCF_001683455.1	RCAD0278	*Maa*	duck	CHN	1-5-9-5-1-7-2-2	2-4-1-3-1-1-2-7/INMV100	8-1-7-4-5-9/ST37*	.GC.	..G.
GCF_003640565.1	HJW	*Maa*	cattle	CHN	1-5-7-5-1-7-2-2	2-3-1-3-1-1-2-7/INMV67	6-1-7-4-5-3/ST21	.GC.	..G.
GCF_009741445.1	DSM44156	*Maa*	hen	n.k.	1-5-7-5-1-7-2-2	2-3-1-3-1-1-2-7/INMV67	6-1-6-4-5-3/ST20	.GC.	..G.
GCF_020735285.1	FDAARGOS_1607	*Maa*	n.k.	GER	1-5-7-5-1-7-2-2	2-3-1-3-1-1-2-7/INMV67	6-1-6-4-5-3/ST20	.GC.	..G.
GCF_020735405.1	FDAARGOS_1606	*Maa*	n.k.	GER	1-7-5-5-1-7-2-2	3-2-1-3-1-1-2-7/NA	7-1-7-4-5-3/ST24	.GC.	..G.
GCF_021183805.1	FDAARGOS_1609	*Maa*	n.k.	GER	1-5-7-2-1-7-2-2	2-3-1-3-1-1-2-7/INMV67	6-1-7-4-5-3/ST21	.GC.	..G.
GCF_021183845.1	FDAARGOS_1608	*Maa*	n.k.	GER	1-5-NA-5-1-7-1-2	2-NA-1-3-1-1-2-7/NA	7-1-7-4-5-3/ST24	.GC.	..G.
GCF_032907725.1	Jb0769	*Maa*	pigeon	GER	1-5-9-5-1-7-2-2	2-4-1-3-1-1-2-7/INMV100	6-1-7-4-5-10/ST22	.GC.	..G.

*CHN *China, *FRA* France, *GER* Germany, *Maa* *Mycobacterium avium* subsp. *avium*, *Mas* *Mycobacterium avium* subsp. *silvaticum*, *NA* *in silico* no analysis possible, *n.k *not known, *UK* United Kingdom

^1^MIRU1, MIRU2, MIRU3, MIRU4, VNTR-25, VNTR-32, VNTR-259, MATR9

^2^VNTR-292, VNTR-X3, VNTR-25, VNTR-47, VNTR-3, VNTR-7, VNTR-10, VNTR-32

^3^*recF, aspB, gnd1, lipT1, pepB,'est'*, *ST37 based on the 5 gene profile by Kolb et al., 2014

^4^Duplex high-resolution melt real-time PCR detecting subspecies specific SNPs in the *yoaK* gene for membrane protein YoaK and the *aspB* gene for aminotransferase, dots indicate identical nucleotides as in the *Mas* 22MA0766 sequence

The two variable sites that were selected by Ronai et al., [21] for a subspecies specific duplex high-resolution melt analyse real-time PCR were also tested for sequence identity and specificity. Here, the sequences were shown to be subspecies specific, i.e. all *Mas* showed ATTA and ACCT sequences, in contrast to the *Maa* strains.

#### Average nucleotide identity (ANI) and genome structure comparison

For comparing the genome of strain 22MA0766 to other *M. avium* species, NCBI’s BioSample database was queried for *Mas* strain data. At the time of writing, only five BioSample entries for *Mas* existed, of which only two were connected to sequencing data (SAMEA1707626, SAMN02470590). The *Mas* reference strain ATCC49884 was the only publicly available genome sequence (GCA_000504975.1), deposited at GenBank, but suppressed in RefSeq database, due to many frameshifted proteins. Also, this genome was highly fragmented (808 contigs). The only sequencing raw data (SRA acc.no. ERR266517 and ERR248977) was from strain MASMRI001 [14], isolated in the United Kingdom. As the available *Mas* genome sequence of strain ATCC49884 had to be excluded from the ANI analysis, due to its fragmentation status, the genome of MASMRI001 was assembled from the Illumina raw data using the SRA data ERR266517. This assembly met the quality criteria for ANI analysis (see above), as it consisted of 65 contigs with an N50 value of 171,759 bp. Thus, the MASMRI001 assembly was included in the further analyses.

**Fig. 5:**
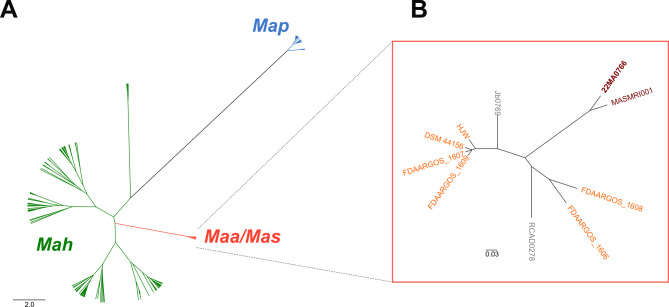
Clustering of *M. avium* strains based on pairwise ANI difference calculation. **A** all *M. avium *strains; **B** only strains of *Maa*/*Mas* complex. *Mas* strains are coloured red, *Maa* strains in orange. Strains for which no subspecies was given in GenBank are coloured grey

Overall, 293 *M. avium* assemblies were downloaded. This set of genomes comprised 185 *Mah*, 59 *Map*, seven *Maa* and one *Mas* genomes as well as 41 *M. avium* strains, for which no subspecies was given (Supplementary Table S3). Of these, 173 passed the quality check and were used for pairwise ANI calculation. The subsequent cluster analysis (Fig. 5A) showed that the *Map* strains formed a group distinct from the other *M. avium* subspecies. In contrast, *Mah* strains were located on several branches and formed various smaller clusters. The *Maa* and *Mas* genomes clustered together and were highly similar. In order to obtain a higher level of resolution, the ANI analysis was repeated for the *Maa*/*Mas* group only (Fig. 5B). *Mas* strains 22MA0766 and MASMRI001 were located at the same branch and shared 99.98% genome identity. However, the difference to *Maa* strains was smaller than 0.12% based on the ANI analysis (Table 2). For comparison, the in silico DNA-DNA hybridization percentage was determined, which confirmed the high identity between 22MA0766 and MASMRI001 (Table 2). In this analysis, the difference to the *Maa* strains was slightly higher (0.9–1.1%).

**Table 2:** Genome identity of 22MA0766 to *Mas* and *Maa* genomes, based on the average nucleotide identity (ANI) and the digital DNA-DNA hybridization percentage (GGDC)

**NCBI RefSeq or SRA accession no.**	**Strain**	**Sub-species**	**Identity with strain 22MA0766**	
			**ANI [%]**	**GGDC [%]**
ERR266517	MASMRI001	*Mas*	99.979027	99.70
GCF_001683455.1	RCAD0278	*Maa *	99.885422	99.00
GCF_003640565.1	HJW	*Maa *	99.890396	98.90
GCF_009741445.1	DSM44156	*Maa *	99.879395	98.90
GCF_020735285.1	FDAARGOS_1607	*Maa *	99.896690	98.90
GCF_020735405.1	FDAARGOS_1606	*Maa *	99.894928	98.90
GCF_021183805.1	FDAARGOS_1609	*Maa *	99.892929	99.10
GCF_021183845.1	FDAARGOS_1608	*Maa *	99.906494	99.00
GCF_032907725.1	Jb0769	*Maa *	99.882309	98.90

**Fig. 6:**
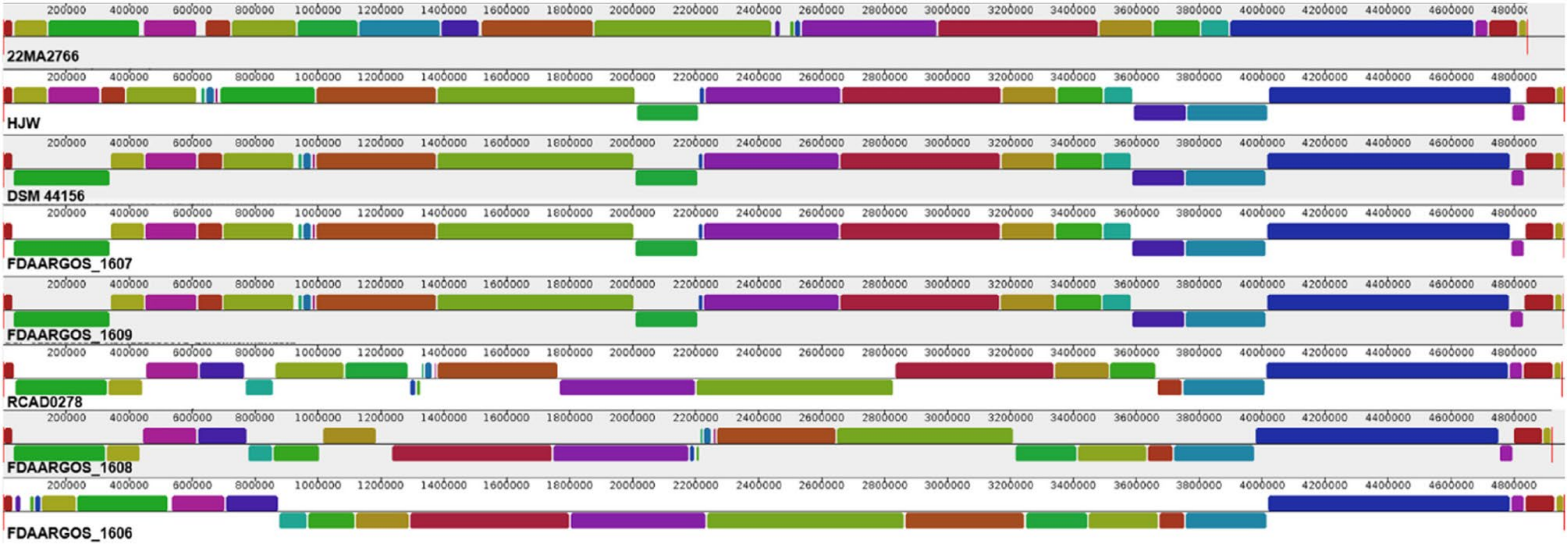
Mauve alignment of 22MA0766 and complete *Maa* assemblies

Complete assemblies of these closely related strains were aligned for investigating difference in the genome structure. Strain 22MA0766 was the only *Mas* strain included in this analysis, as other *Mas* assemblies were incomplete and the order of the contigs elusive. It was found, that there were large genome rearrangements and inversions, even among *Maa* strains (Fig. 6).

All of the *Maa* genomes were larger than that of 22MA0766 (78.6 to 120.3 kb), which could be a general characteristic of *Mas*, as the two incomplete *Mas* genome assemblies also were smaller in size (4.71 Mb and 4.79 Mb). However, as the incomplete assemblies lack an unknown number of bases, this cannot be definitively resolved.

#### Metabolic pathways and gene content

As the genomic similarity was found to be extremely high and differentiation of the two subspecies is primarily based on phenotypic differences, the potential metabolic capabilities of the *Maa* and *Mas* strains were investigated. In the two *Mas* strains 404 predicted, functional pathways were identified, whereas for the seven *Maa* strains 406 to 408 pathways could be found (Supplementary Table S4). There were two pathways, that were present in all *Maa* genomes, but not in *Mas*: the biosynthesis of ergothioneine (ergothioneine biosynthesis II) and the carotenoid neurosporene, respectively. Ergothioneine biosynthesis pathway I was incomplete (80% completeness), while complete in *Maa*.

The comparison of *Mas* and *Maa* strains on the gene level showed, that all shared 94.7% of genes (*n* = 4,387). Only 17 genes were identified, that were present in all *Maa* strains but not in *Mas*. However, some of these missing genes had functional homologues in *Mas*. In turn, solely five genes were unique to the two *Mas* strains, four of which were predicted as hypothetical and the other one as encoding a dihydrodipicolinate reductase family protein. In agreement with the metabolic pathway analysis, genes for carotenoid synthesis were missing in *Mas*.

**Fig. 7:**
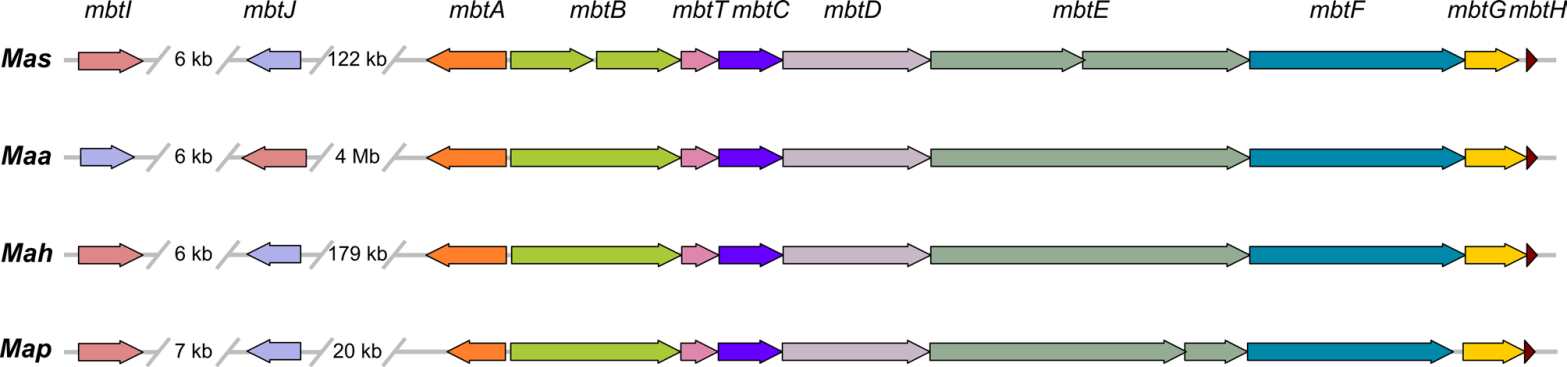
Schematic representation of the *mbt*−1 locus in *Mas* (22MA0766), *Maa* (RCAD0278), *Mah* (104) and *Map* (K-10). Homologous genes are coloured identical. Gene names are given above

As growth of 22MA0766 on medium lacking mycobactin was delayed, a closer look was taken on the genes required for mycobactin synthesis (*mbt*−1 locus) by comparing them to other *M. avium* strains with mycobactin dependence (*Map*) or independence (*Maa* and *Mah*). For better comparability, the genomes of the *Maa*, *Mah* and *Map* strains used for comparison were re-annotated. The arrangement of genes in the mycobactin synthesis cluster *mbtA* to *mbtH* was conserved in all four strains (Fig. 7). However, two gene disruptions were found in *Mas* compared to *Maa* strain RCAD0278, as *mbtB* as well as *mbtE* comprised two open reading frames each in 22MA0766. Additionally, the product of *mtbG* was truncated at the C-terminal end in 22MA0766 (368 amino acids [aa]) compared to RCAD0278 (428 aa). In contrast, MbtA, MbtC, MbtD, MbtH and MbtT were identical in *Mas* and *Maa*, but differed from the other two subspecies. In *Map* K-10, *mbtA* and *mbtF* were truncated and *mtbE* was disrupted, yielding two open reading frames. While in *Mas* the *mtbE* disruption resulted in two predicted proteins of similar size (1,148 aa and 1,059 aa), the predicted MbtE proteins of *Map* were of different sizes (1,759 aa and 431 aa). The genes *mbtI* and *mbtJ* were separated from the rest of the *mbt*−1 locus. In *Maa* the distance was particularly large (~ 4 Mb) and there was an inversion relative to *mtbA-H*. Nevertheless, the protein sequences for MbtI and MbtJ were identical in *Mas* and *Maa*. MbtJ was 29 aa shorter and differed in its sequence in *Mah* and *Map*, whereas *Mah* MbtI was identical to that of *Maa*/*Mas*. In *Map*, MbtI was of identical length, but showed two amino acid changes.

## Discussion

Of the *M. avium* subspecies, *Mycobacterium avium* subsp. *silvaticum* is the one that is the least reported and least genetic information is available. It has been known as ‘wood pigeon mycobacteria’ before definition of the subspecies [13]. However, its host spectrum is not restricted to - mainly wild - avian species, as *Mas* has also been isolated from mammals [52, 53]. Here, we reported the isolation of *Mas* strain 22MA0766 from an Egyptian goose in Germany and present the first closed whole genome sequence of a *Mas* strain.

The genome of 22MA0766 comprised a single circular chromosome of about 4.84 Mb, without any plasmids. Due to the lack of public available *Mas* whole genome sequences, nothing is known about the genomic diversity of this subspecies. However, we did compare the genome structure of 22MA0766 to genomes of closely related *Maa* strains and found a high heterogeneity, i.e. numerous re-arrangements and inversions, even between the *Maa* strains. This is in line with findings for *Map* and *Mah*, in which genome re-arrangement and recombination events are commonly found [54, 55].

It has long been debated whether *Mas* and *Maa* should be combined into a single subspecies of *M. avium* [18, 56]. Their high congruence of 98% in DNA-DNA hybridisation experiments has been acknowledged [18, 53], which corresponds to the exceptionally high average nucleotide identity of more than 99.8% found between *Maa* and *Mas* in the present study, although our ANI and GGD results were slightly lower than those from Tortoli et al. [18].

Classic PCR-based typing methods identified allelic profiles that were considered typical for *Mas*, i.e. INMV99, MLST ST26/27 and nucleotide variances in *yoaK* and *aspB* [21, 46, 57], which was also true for 22MA0766. The discriminatory power of such methods is relatively low compared to the possibilities offered by whole genome-based analyses.

The current gold standard for species delimitation is the determination of the average nucleotide identity (ANI) between genomes, usually using a cut-off between 95% and 96%. In mycobacteria, the ANI was found to be particularly high, i.e. 98.82% for *M. avium* strains, indicating a high level of genome conservation between *M. avium* subspecies [41, 58]. Further, numerous studies showed that *Mas* was located in the same cluster as *Maa* strains when analysing nucleotide differences in the core genome region [59, 60]. The high degree of genomic similarity identified by ANI analysis indicates that *Maa* and *Mas* should/could be considered as variants of a single subspecies, as already proposed by several authors [18, 56, 60]. However, the high genetic homology despite the large spatial and temporal difference between the isolation of the individual *Mas* strains (22MA0766: 2022 in Germany; MASMRI001: year unknown in UK; ATCC 49884: 1978 in France) could be interpreted as indicator of the subspecies-specificity and indicates an exceptionally low mutation rate of this pathogen. The lack of high-quality sequencing data impedes the detailed differentiation of *Maa* and *Mas* at the genomic level, as it is hardly possible to differentiate whether observed differences are strain- or subspecies-specific. Tortoli and colleagues suggested to summarize *Maa*,* Mas* and *Mah* as variants into one subspecies and to keep subspecies *Map* separated based on ‘epidemiology, pathogenicity, evolution and physiology’ [18]. It is difficult to identify specific pathogenicity or epidemiology traits for *Mas* as only few data are available. The former description as ‘wood pigeon mycobacteria’ [13] implicates the occurrence of *Mas* solely wild birds. Beside wild birds, in the recent years *Mas* strains were also isolated from mammals, like horses [52], red deer, red foxes and wild boar [15]. However, more isolates with at least information on the origin, clinical signs in the affected host and whole genome data are required for a clear classification as subspecies or variant of a subspecies. Likewise, the quality of metadata deposited in public databases is of utmost importance. Incorrect or incoherent strain designations can lead to confusion and incorrect conclusions in later studies. Here, we encountered incoherences with the presumed *Maa* strain HJW, which was described as *Maa* in the original publication [61], while deposited as *Mas* or *M. avium* in NCBI (CP028731.1, SAMN08892076). Based on our analyses, the strain HJW belongs indeed to *Maa*.

Insertion sequences (IS) are widely distributed in *M. avium* and are used for subspecies identification. Therefore, most IS elements found in 22MA0766 were expected, like IS*1311* (present in all *M. avium* complex members) or IS*1245*, IS*1601* and IS*Mav1* (present in *Maa*,* Mas*,* Mah*) [12]. Although IS*902* was initially thought to be *Mas-*specific [62], it was later on shown that IS*901* and IS*902* share 99% nucleotide identity and might be one element [63]. Interestingly, the sequences detected in 22MA0766 were hybrid sequences between the published sequences of IS*901* [64] and IS*902* [62], as they showed specific nucleotides from both elements. This result confirms the unsuitability of IS*902* as a *Mas*-specific marker in diagnostic approaches.

The prediction of metabolic pathways in *Mas* and *Maa* showed, that both subspecies are highly similar in their genetic traits. There were two pathways, that were found in all *Maa* but missing in *Mas*: synthesis of ergothioneine (pathway II) and neurosporene.

Ergothioneine is a low-molecular-weight thiol, which plays a role in oxidative stress response and nutrient starvation [65, 66]. In *M. tuberculosis*, lack of ergothioneine impeded long-term infection of murine macrophages, due to higher stress levels caused by bactericidal molecules [65]. Thus, a role of ergothioneine in mycobacterial virulence has been suggested [67]. In *Mas*, the lack of ergothioneine synthesis could lead to a narrower host range and fewer fatal infections, as the bacterium has a lower stress tolerance to the host cell environment. As there is a lack of data on the virulence of *Mas*, the potential impact of missing ergothioneine synthesis on its fitness in comparison to *Maa* remains elusive.

Neurosporene is a carotenoid pigment which has been found in several bacteria [68–70] but little is known about the function of this pigment in mycobacteria. The heterologous expression of neurosporene and its converted product, lycopene, from *M. aurum* in *Escherichia coli* increased the transformant’s resistance to ultraviolet radiation [68, 70]. The pangenome analysis as well as the metabolic pathway prediction both indicated that neurosporene and thus also lycopene synthesis is lacking in *Mas*, whereas the *Maa* strains could be able to produce these compounds. In general, carotenoids are considered to increase antimicrobial resistance and virulence as they can protect bacterial cells against oxidative stress in host cells [71, 72]. However, Stormer and Falkinham [73] investigated pigmented *Mah* strains and unpigmented natural mutants of these and made opposing observations: the unpigmented variants were more tolerant to antibiotics and heavy metal salts than their pigmented parental colonies. Also, pigmented colonies grew more rapidly on most media, but did not grow on agar with Tween 80 as sole carbon source. In *Map,* pigmented and unpigmented variants are known, although pigmentation often correlates with the ovine *Map* type, that generally grows very slowly [74]. Thus, the role of carotenoids in *M. avium* subspecies remains elusive. However, analysing metabolic pathways, for both, *Maa* and *Mas*, the biosynthesis of mycobactin was predicted as present. Phenotypically it was shown that 22MA0766 required mycobactin supplementation in egg-containing media, while in egg-free broth the strain grew also without mycobactin, but mycobactin supplementation increased the growth. Interestingly, similar mycobactin in-/dependent growth characteristics were shown for *Map* isolates from sheep [75]. Mycobactin is a siderophore (iron chelator), that plays an important role in iron uptake [48]. While many reports describe *Mas* as mycobactin-dependent [76], the *Mas* strain 22MA0766 was not strictly mycobactin-dependent as it did grow in 7H9 broth without mycobactin. Similarly, Thorel et al. 1990 [13] observed that *Mas* growth is hampered on egg-based media and promoted by supplementation of mycobactin.

The *mbt* operon, encoding 11 proteins MbtA to MbtJ and MbtT involved in mycobactin synthesis, was first described in *M. tuberculosis* [77]. Two *M. avium* subspecies, *Map* and *Mas*, require supplementation of medium with mycobactin for optimal growth in vitro. It has been shown that the arrangement of the genes required for mycobactin synthesis differed in *Maa* and *Map* from the presumable ancestor cluster in MTC [48], i.e. *mbtI* and *mbtJ* were separated from the other genes of the cluster. This was also the case in *Mas*, as shown in the present study. In *M. smegmatis*, *mbtB*,* mbtE* and *mbtG*, among others, are required for mycobactin synthesis [48]. The product of *mtbG*, a lysine hydroxylase, was truncated in *Mas*, compared to *Maa*, which could impair its functionality. Further, for *mbtB* and *mbtE* two consecutive open reading frames each were annotated in 22MA0766, yielding two smaller protein products. MbtB and MbtE are required for inclusion of amino acid residues in the siderophore [78]. In *M. tuberculosis*, the deletion of *mbtB*, encoding an acyl carrier protein, lead to mutants with restricted growth on iron-limited medium [79]. It can be assumed that the truncation of MbtB and MbtE in *Mas* impedes the proteins’ functions, hampering mycobactin synthesis and leading to mycobactin dependence. Most of the other MBT proteins were identical to those of *Maa* and thus can be considered functional. In contrast, in *Map* K-10, the mycobactin dependence was the result of frameshift mutations leading to pseudogenes of *mbtA*, *mbtE* and *mbtF*. However, different variations in *mbtE* were found in *Map* strains [80]. Thus, the mycobactin dependence of *Map* and *Mas* results from gene truncation and disruption in both subspecies, although the *mbt* locus genes are affected differently. The dysfunctionality of MtbE appears to be the only common feature.

## Conclusion

The first complete genome sequence of a *Mas* isolate presented in this study closes a knowledge gap. Although the collection of further *Mas* genome data is necessary, the existing data set already enables a deeper analysis of *M. avium*. The found differences in growth requirements and the *Mas* gene content compared to the closest relative *Maa* seem to be stable and independent of spatial (France, UK, Germany) and temporal (> 40 years) differences on their isolation. However, further investigations of potential *Mas* strains based on both phenotype and genotype are required to definitively determine the position of this subspecies within the *M. avium* phylogeny. The data presented in this study cannot rule out the necessity of merging the two subspecies, *Maa* and *Mas*, into one. Further epidemiological and genomic evidence is required.

## Data Availability

Supporting files include Tables S1 to S4 and Figures S1 and S2 are accessible via the Zenodo repository (10.5281/zenodo.15363855). The raw sequences and assembled genomes are deposited in the European Nucleotide Archive (project no. PRJEB87763).
